# False Beliefs about Mathematics in Spanish Pre-Service Teachers and Relations with Math Anxiety

**DOI:** 10.3390/ejihpe14070127

**Published:** 2024-06-28

**Authors:** M. José Justicia-Galiano, Santiago Pelegrina

**Affiliations:** Department of Psychology, University of Jaén, 23071 Jaén, Spain; mgaliano@ujaen.es

**Keywords:** false beliefs, math myths, math anxiety, pre-service teachers, mathematics

## Abstract

Teachers may hold beliefs about how students learn mathematics and about the subject itself. However, some of these beliefs, often referred to as “math myths”, may be oversimplified ideas that appear valid but lack solid scientific evidence. This study was aimed to investigate the prevalence of math myths among Spanish pre-service teachers and compare it with the degree of support for these myths reported in previous studies; investigate the possible underlying structure of a set of false beliefs about math; and determine the relationships of certain math misconceptions with math anxiety. A total of 212 Spanish pre-service teachers were administered questionnaires about math myths and about general and math anxiety. The results showed prevalence patterns of each math misconception similar to those in previous studies. False beliefs about math showed weak or negligible correlations with each other and did not exhibit a discernible underlying structure. Furthermore, math anxiety was related to the belief that some individuals are perceived to possess a “math mind” while others do not. Our results suggest diverse origins for the development of different math myths, rather than the myths being the consequence of a single underlying factor. Finally, the endorsement of certain beliefs about who can do mathematics may contribute to negative emotions towards mathematics.

## 1. Introduction

Teachers maintain beliefs about how students learn and about the subjects they teach [1]. While many beliefs are accurate, some are oversimplified ideas or appear valid but lack solid scientific evidence. Certain false beliefs, often referred to as myths, are prevalent in various fields, including psychology [2,3,4], educational psychology [5], neuroscience [6,7,8,9,10,11,12], and mathematics [13,14,15]. False beliefs can also be found in the field of education. Teachers may hold some beliefs because they seem logical or are based on their own practical experiences, even if they are not based on evidence [5,16]. It has been argued that teachers’ beliefs may influence their teaching practices, leading them to use pedagogical methods that are not scientifically validated [6,11,16,17]. Furthermore, teachers may have the capacity to transmit their beliefs to their future students [18]. Therefore, it is worth investigating false beliefs in education, as they can influence the teachers’ approach to teaching and decision making.

### 1.1. False Beliefs about Mathematics

Teachers also develop beliefs about mathematics that, similar to other beliefs, may potentially shape their teaching practices and influence their students’ math achievement [19,20]. As with neuromyths, some of these beliefs may not be based on scientific evidence. Phelps-Gregory et al. [15] examined a set of 12 false beliefs that were originally proposed by Kogelman and Warren [21] and subsequently investigated in several samples of US students [13,14,15] (see Appendix A). For descriptive purposes, Frank [14] grouped them into the following three categories: who can do mathematics, how mathematics is learned or practiced, and the nature of mathematics. These three categories of false math beliefs are briefly outlined below. A more detailed account of each myth and the counter-arguments is offered by Phelps et al. [15].

The first group of ideas pertains to who can do mathematics. A common belief is that only individuals with a natural aptitude for mathematics can excel in the subject (Item 1), suggesting that mathematical learning depends on innate ability; however, any student has the potential to achieve proficiency in mathematics with the application of effective strategies and appropriate instructional support. Another misconception is that male students inherently outperform female students in math (Item 7); however, there is evidence that both genders may perform equally well in this subject (see Ref. [15] for a more detailed account).

A second group of beliefs relates to the learning and practice of mathematics. One misconception is that understanding the process behind an answer is crucial (Item 3); however, it is not always necessary to understand the method that led to the answer. Also, it is often claimed that there is a single method that is superior for solving a mathematical problem (Item 5); however, a solution may be reached through different approaches, none of which are inherently better than the others. Similarly, another myth is that the goal is always to obtain the exact answer (Item 8); however, this notion undervalues estimation, where exact answers are not always necessary. Finally, there is a belief that relying on one’s fingers for calculations is indicative of inadequacy (Item 12); however, finger counting is a developmental stage in arithmetic learning and may help people to make associations between numbers and spatial concepts (see Ref. [15] for a more detailed account).

Finally, another set of false beliefs entails a reductionist view of the nature of mathematics. One belief is that mathematics requires logic and not intuition (Item 2); however, mathematical knowledge may be based on two complementary modes of understanding: intuition, which is more immediate, and reasoning, which is more reflective. Other misconceptions are that math involves memorizing (Item 10) and that there exists a “magic key” for doing mathematics (Item 4). While memory plays a role in mathematical learning, it is not the only determinant; conceptual understanding and reasoning are also essential (see Ref. [15] for a more detailed account).

A relevant question is whether the different math beliefs can be grouped around the same construct or whether they are independent. Most studies on neuromyths have not investigated whether the items included in questionnaires form a coherent underlying construct, and the few studies that have addressed this issue [8,22] have found little consistency between the items included in these measures. Regarding mathematics beliefs, it is also unclear whether they can be grouped into coherent factors. This was one of the objectives of the current study.

Another important question is whether beliefs are consistent across cultures. Many beliefs are shared by people from different cultural backgrounds. For instance, in the field of neuroscience, specifically its application to education, neuromyths are widespread among teachers from numerous countries in several continents (see Ref. [23] for a review). For example, Varea et al. [4] found that the most prevalent myths in psychology in Spain coincide with those found in the US; however, some misconceptions that were common among students in the US were hardly seen at all in Spain. Regarding studies on teachers’ false math beliefs, we only found studies of US students [13,14,15]. Given the lack of research on mathematical myths in different contexts, it may be useful to examine the prevalence of these false beliefs in other countries.

### 1.2. Math Beliefs and Math Anxiety

Some false beliefs about who can do mathematics may be associated with negative emotions towards mathematics, particularly math anxiety. Math anxiety refers to the negative emotions, worry, and nervousness that individuals feel in math situations. It encompasses a state component, which reflects anxiety in specific mathematical situations, and a trait component, i.e., an acquired and relatively persistent tendency to view math-related situations as threatening [24]. Math anxiety is negatively associated with mathematics performance [25], and this relationship has been consistently observed across countries [26]. Previous research involving teachers or prospective teachers focusing on trait-math anxiety has demonstrated a negative relationship between math anxiety and various aspects of mathematics teaching [27] or math performance [28]. Additionally, math-anxious teachers often utilize more traditional teaching approaches, emphasizing, for instance, rote practices [29,30].

It is possible that math anxiety is also related to certain beliefs about who can do mathematics [31]. Specifically, the notion that mathematical ability is predetermined or fixed may induce negative emotions towards mathematics in individuals with a history of poor performance in the subject. Indeed, some authors have claimed that some beliefs about mathematics contribute to mathematics anxiety [31]. Another false belief potentially associated with math anxiety is the perception that male students outperform their female counterparts in math. If female students endorse the belief that women are less competent in math, they may exhibit heightened levels of math anxiety [32,33]. An aim of the current study was to examine the relationship between beliefs about who can do math and negative emotions towards this subject among pre-service teachers.

### 1.3. Present Study

The present study, building on previous research by Phelps-Gregory et al. [15], was aimed to investigate the prevalence of false mathematic beliefs among Spanish pre-service teachers. This would allow us to compare the prevalence of false beliefs across studies in different cultural contexts. Given the high acceptance of some myths about mathematics noted in previous studies [13,14,15], we expected to find comparable levels of support for these math misconceptions among pre-service teachers in Spain.

In addition, we sought to determine some psychometric properties of the false beliefs questionnaire developed by Frank [14]. To our knowledge, neither the consistency nor the structure of the questionnaire has been tested. Testing these characteristics should be performed prior to obtaining global scores for the items comprising a scale. A strong relationship among items would suggest that there is a common factor that makes it easier for people to hold misconceptions about mathematics, while a weak association might suggest that diverse factors contribute to each belief.

Finally, we aimed to determine the extent to which certain beliefs about who can do mathematics are associated with emotional factors. We anticipated that math anxiety would be related to the endorsement of the idea that some people have a “math mind” whereas others do not and, in female participants, to the belief that males outperform female students in math.

## 2. Materials and Methods

### 2.1. Participants

The initial sample consisted of 266 pre-service teachers (primary education) in their first year. They volunteered in the screening phase of a study focused on individual differences in math anxiety. This phase consisted of completing questionnaires on math and general anxiety among other measures not included here. In total, 44 participants were excluded because they failed to respond correctly to two control questions aiming to verify that participants read and understood the questions before answering them. Thus, the final sample was made up of 212 participants (84 males and 128 females) aged between 17 and 40 years (M = 19.85, SD = 2.6). The study was approved by the Ethics Committee of our university. Participants provided written informed consent before participating in the study.

### 2.2. Measures

False beliefs about math. The questionnaire developed by Frank [14] (see Appendix A), with the response format used by Phelps et al. [15], was adapted to measure beliefs about mathematics. The items were translated into Spanish by two native Spanish speakers with high English proficiency. Any disagreements in translation were resolved through discussion to create a unified version. Finally, an independent expert reviewed the final Spanish version. The instrument included 12 items that, according to Phelps et al. [15], can be grouped into three categories: (a) myths about who can do mathematics (Items 1 and 7; e.g., some people have a math mind, and some do not), (b) myths about how mathematics is learned or practiced (Items 3, 5, 6, 8, 9, and 12; e.g., you must always know how you got the answer), and (c) myths about the nature of mathematics (Items 2, 4, 10, and 11; e.g., math requires logic, not intuition). Agreement or disagreement with each of the 12 statements was indicated using a 5-point Likert scale (1 = not true, 3 somewhat true, 5 = very true).

Math anxiety. The Abbreviated Math Anxiety Scale (AMAS) [34], Spanish version [35], is a 9-item measure of anxiety in learning or instructional situations (e.g., listening to a lecture in math class), as well as evaluation contexts (e.g., being given an assignment of completing many difficult math exercises due for the next class meeting). Responses are provided via a 5-point Likert scale (1 = not nervous at all; 5 = very nervous). Scores on the AMAS range from 9 to 45, with higher scores indicating greater math anxiety. The Cronbach’s α value in the current study was 0.87.

General anxiety. The State-Trait Anxiety Inventory—Trait version (STAI) [36], Spanish adaptation [37], was employed to measure general anxiety. This questionnaire comprises 20 items (e.g., I worry too much over something that really does not matter) that participants rate on a 4-point Likert scale (0 = almost never, 3 = almost always) based on how they feel “in general”. Scores range from 0 to 60, with higher scores indicating higher trait anxiety. The inventory demonstrated high internal consistency in the current study (Cronbach’s α = 0.90).

### 2.3. Procedure

Participants completed all the measures through an online questionnaire administered in class at the conclusion of a developmental psychology lecture during the first semester. The assessment lasted about 15–20 min.

### 2.4. Data Analysis

An exploratory factorial analysis was conducted on the data to investigate the structure across the items included in the questionnaire. The number of factors to extract from the EFA was based on parallel analysis and Velicer’s minimum average partial (MAP) test. Then, a factor analysis was conducted using the psych package [38] for R software (version 4.0.2) [39].

To assess whether the item scores in this study were consistent with trends observed in previous research using the same questionnaire, Pearson correlations were calculated between the scores of the 12 items in the present study and those from previous studies. The 95% confidence intervals (CIs) were obtained using bootstrapping with 2000 resamples.

To gain further insight into global patterns in the prevalence of math beliefs across the different studies, we compared the data from the present study with those obtained by previous studies, as reported by Phelps-Gregory et al. [15]. Following the analytic approach that Ferrero et al. [11] used for neuromyths, we meta-analyzed the proportion of participants in each study who endorsed each statement regarding math.

Furthermore, Spearman’s rank-order correlation coefficients were calculated to assess the degree of inter-item correlation among the math belief items. This analysis was aimed to identify the extent to which scores on each math belief item were related to each other.

Finally, we examined the relationship between math anxiety and two specific beliefs related to who can do mathematics (i.e., Items 1 and 7). A set of multiple regression analyses were performed on scores obtained for each item, with gender and math anxiety scores as predictors. In order to control for general anxiety, the STAI score was also included as a predictor.

## 3. Results

### 3.1. Exploratory Factor Analysis

Horn’s parallel analysis indicated that two factors should be extracted, whereas Velicer’s MAP suggested the extraction of one factor. Thus, solutions for one and two factors were examined. The single-factor solution accounted for 17% of the variance, whereas the two-factor solution using varimax rotation explained 24% of variance. In the latter solution, the first factor included Items 1, 2, 3, 4, 5, 6, 8, and 9, and the second factor included Items 7, 8, 9, 10, and 12, with two cross-loading items (Items 8 and 9). Overall, these results indicate that there is no structure that clearly explains a considerable amount of the variance in the 12 items. In any case, the items appear to be grouped according to their prevalence, with items with higher prevalence belonging to Factor 1 and those that received less support to Factor 2.

### 3.2. Prevalence of Beliefs about Math

**Figure 1:**
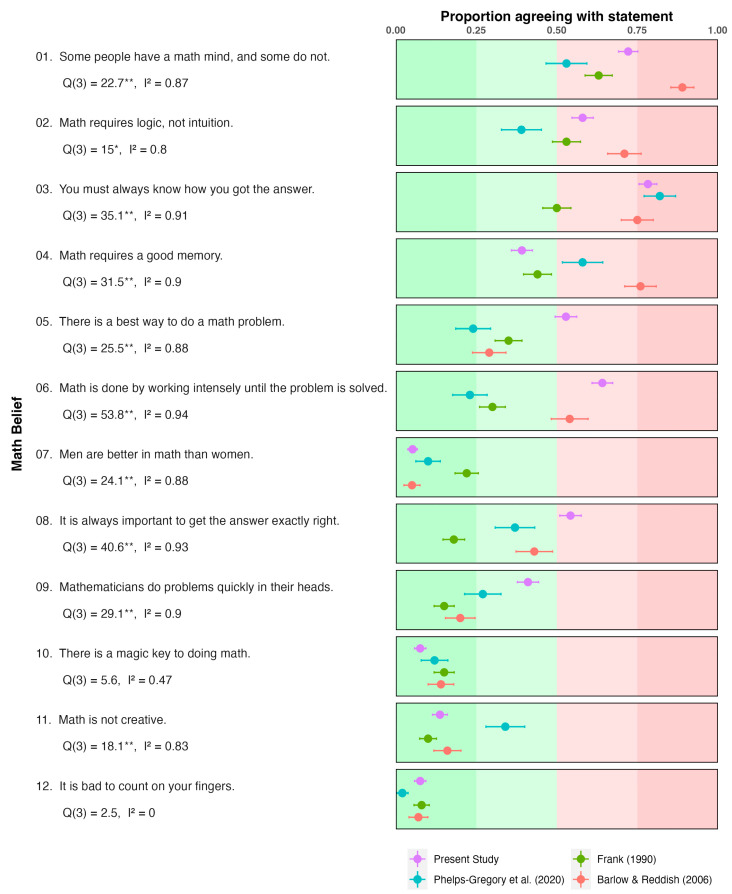
Proportion of participants agreeing with each math myth across different studies. The error bars represent 95% confidence intervals [13,14,15]. Q: Cochran’s Q-statistic; I2: Higgins’ and Thompson’s I2. ** *p* < 0.001; * *p* < 0.05.

Figure 1 shows the percentage of agreement, as well as the score obtained, for each math belief both in the present study and in other studies. In the current study, the most prevalent math beliefs (agreement >50%) were those associated with Item 3 (you must always know how you got the answer), Item 1 (some people have a math mind, and some do not), Item 6 (math is done by working intensely until the problem is solved), Item 2 (math requires logic, not intuition), Item 8 (it is always important to get the answer exactly right), and Item 5 (there is a best way to do a math problem). Thus, participants incorrectly endorsed half of the beliefs presented. In contrast, some beliefs showed very low prevalence (<15%), i.e., those associated with Item 11 (math is not creative), Item 10 (there is a magic key to doing math), Item 12 (it is bad to count on your fingers), and Item 7 (men are better in math than women).

Overall, no gender differences were observed in the endorsement of most beliefs, except for two beliefs with low prevalence: Items 7 (men are better in math than women) and 10 (there is a magic key to doing math). In both cases, male pre-service teachers scored higher than their female counterparts (*p* < 0.05).

### 3.3. Comparison to Prevalence in Previous Studies

To determine whether item scores in this study mirrored trends observed in previous studies using the same questionnaire, Pearson correlations were calculated between the scores obtained for the 12 items in the present study and those from the earlier studies. Strong correlations (*r* > 0.7) were found between item scores observed in the present study and those from Frank [14] (*r* = 0.76, *p* = 0.004, 95% CI [0.49, 0.91]), Barlow and Reddish [13] (*r* = 0.83, *p* < 0.001, 95% CI [0.58, 0.95]), and Phelps-Gregory et al. [15], (*r* = 0.72, *p* = 0.008, 95% CI [0.30, 0.92]). Thus, the rank order of scores for each item was similar across the current and previous studies.

Figure 1 shows a forest plot of the prevalence of math beliefs. Two observations merit consideration. Firstly, a similar trend to that described for correlations can be observed, with some math beliefs showing high prevalence and others evidencing low prevalence across studies. Secondly, there was considerable heterogeneity within each item. For most math beliefs, more than 80% of the variance can be attributed to systematic differences between studies. Only items 10 (there is a magic key to doing math) and 12 (it is bad to count on your fingers) showed low-to-moderate heterogeneity (*I*^2^ < 40%), which can be attributed to the very low prevalence of both beliefs across studies.

### 3.4. Inter-Item Correlations

**Figure 2:**
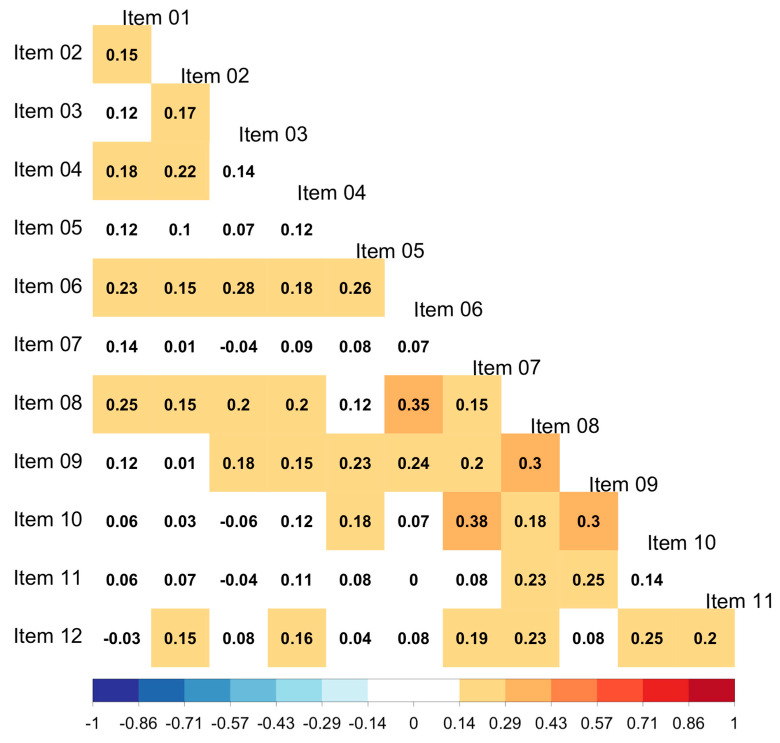
Spearman inter-item correlations. Correlations with a white background are not significant.

Spearman inter-item correlations were computed to determine the extent to which scores for the different math beliefs were related to each other. Figure 2 shows that, overall, the inter-item correlations were rather small, with most of them being below 0.2 and non-significant, suggesting that the items do not represent the same domains

### 3.5. Relationship between Math Beliefs and Math Anxiety

We were also interested in examining the relationship between math anxiety and two specific beliefs related to who can do mathematics (i.e., Items 1 and 7). A set of multiple regression analyses was performed on scores obtained for each item, with gender and math and general anxiety scores as predictors.

Results showed that the endorsement of Item 1 (some people have a math mind, and some do not) was related to math anxiety (β = 0.17) after controlling for general anxiety. The model accounted for a small proportion of the variance (R2 = 0.06; adjusted R2 = 0.04, *p* = 0.005). The standardized regression coefficients are shown in Table 1.

The belief espoused in Item 7 (men are better in math than women) was only related to gender (β = 0.36), as female students endorsed this belief to a lesser extent than male students. No other predictor reached statistical significance (see Table 1). The amount of variance explained was relatively small (R2 = 0.11; adjusted R2 = 0.10, *p* < 0.001). Analogous analyses of male and female subgroups did not show any significant effect (ps > 0.1). Therefore, math anxiety was not related to the belief that men are better than women in math.

## 4. Discussion

The present study investigated the prevalence of false mathematics beliefs among Spanish pre-service teachers. Our results revealed remarkably similar prevalence patterns of math myths across studies from different cultural contexts. Furthermore, false beliefs appear to be independent of each other, and they do not exhibit a clear underlying structure. Finally, regarding beliefs about who can do math, the notion that some people have a math mind while others do not was found to be associated with math anxiety.

### 4.1. Underlying Structure of Items in the Math Myths Questionnaire

A solution derived from the factor analysis indicated that two factors grouped the items according to the degree of support received rather than by the nature or type of belief. Factor 1 encompassed most of the items that received the highest support, while the remaining items were grouped under Factor 2. Similar results were reported in the study of Macdonald et al. [8] on neuromyths, where the first factor mainly comprised more highly endorsed beliefs, while the other factors included items with less support. Each factor is made up of a mixture of different types of beliefs according to the classification of Phelps et al. [15]. For instance, the first factor encompasses items about who can do mathematics (e.g., some people have a math mind, and some do not), how mathematics is learned (e.g., you must always know how you got the answer), and the nature of mathematics (e.g., math requires logic, not intuition). Consequently, it is challenging to identify a common theme for the first factor; instead, it appears to capture the most prevalent misconceptions about mathematics tested in the present study.

It is important to note that the identified factors accounted for explained a small amount of the variance, indicating that they do not conform to an underlying structure. This observation is further reflected in the small and non-significant correlations observed between the levels of support for the different beliefs. Thus, individuals might endorse specific beliefs without necessarily aligning with others, suggesting that math beliefs exhibit a degree of independence from each other. Howard et al. [22] also found little consistency between the items included in a neuromyth questionnaire. If there is no underlying structure for a scale, there is little justification for aggregating item responses in a global index.

Therefore, our findings point to diverse origins for the development of different beliefs, rather than one underlying factor that predisposes students to hold various false beliefs about math. Given that our participants were at the beginning of their teacher education studies, it would be premature to attribute their false beliefs to biased interpretations of educational materials or curriculum content. Rather, these beliefs may originate from personal experiences as students, from notions transmitted or shared among students during earlier stages of education, or even from misinterpretations or distortions of scientific facts (see Ref. [16] for neuromyths). Hence, it would be worthwhile to explore the origin of the misconceptions about mathematics, how they manifest in primary and secondary education, and whether they persist into higher education among student teachers.

### 4.2. Comparison of Prevalence across Studies

Overall, the pattern of agreement for the different beliefs in this study is analogous to those found in the US and over different years. The rank order of beliefs in our study is comparable to those found in previous studies using the same questionnaire [13,14,15]. In general, several items (1, 2, and 3, as reflected in Figure 1) consistently received the highest levels of endorsement across all studies, while beliefs associated with other items (7, 10, 11, and 12, see Figure 1) were generally poorly supported. Notably, in the context of neuromyths, Howard-Jones et al. [17] and Ferrero et al. [11] also noted remarkable similarities in the prevalence patterns of some misconceptions in countries with very different cultures. Furthermore, beliefs about mathematics appear to persist over time, mirroring the findings for neuromyths (see Ref. [40]).

Despite the consistency in the rank order of support for the different beliefs, there is a certain degree of heterogeneity across countries in the level of agreement with specific beliefs. In our case, three beliefs (associated with Items 5, 6 and 8) received more support compared to previous studies, while one belief (Item 4) received less support. This type of variability across countries has also been reported for neuromyths [11]. In summary, the relative prevalence of the different false beliefs is comparable across different studies, although there are differences in the degree of support of particular beliefs.

### 4.3. Relationship of Math Beliefs with Math Anxiety

We were particularly interested in exploring potential associations between support for mathematical beliefs related to who can do mathematics (Items 1 and 7) and math anxiety. The association with the first belief, i.e., that “some people have a math mind, and some do not”, is particularly intriguing, suggesting that individuals with a fixed mindset tend to exhibit higher levels of mathematical anxiety. This finding aligns with the recent results obtained by Gonzalez-DeHass et al. [41] in undergraduate students (but see Ref. [42] for a report of the absence of such a relationship). Students with a fixed mindset adhere to the notion that skills and abilities are immutable, with brilliance and talent in mathematics perceived as innate and biologically predetermined traits [43]. This belief may lead to a lack of motivation, effort, and persistence in the student and the avoidance of mathematics-related careers, which in turn may result in both poor academic performance and feelings of math anxiety.

The stereotyped belief that men are better than women in mathematics was not endorsed by the pre-service teachers. This finding aligns with existing research indicating that secondary school students have an egalitarian view of men’s and women’s mathematical abilities [32]. Nonetheless, even with this low support, males tended to hold the stereotyped belief to a greater extent. The very low prevalence of this belief may explain why we found no relationship between this belief and math anxiety in female participants.

### 4.4. Implications and Limitations

A logical implication of the present study is that efforts should be made to eliminate false beliefs, particularly those that are highly prevalent. It has been proposed that some misconceptions about psychology, and specifically about mathematics, could be detrimental to learning or teacher practice [15,44]. However, in the case of neuromyths, some studies have failed to find such an association. For example, Horvath et al. [22] did not observe differences in the levels of support for neuromyths between a group of award-winning teachers and a group of trainee and non-award-winning teachers, and Krammer et al. [45] found that, in general, pre-service teachers’ academic grades were not related to their acceptance of neuromyths. Thus, these studies raised doubts about whether maintaining certain neuromyths negatively impacts teaching or academic achievement. We contend that further research is necessary to verify the specific relationship between false beliefs about mathematics and math learning or teacher practice.

In any case, it appears prudent to address false beliefs about mathematics because teachers should have a sound knowledge of the subjects they teach, and such knowledge should be based on evidence [45]. This objective could be achieved by incorporating such content into the curriculum of prospective teachers [11,13,15]. Refutational techniques have proven effective at reducing belief in psychological myths, and they could also be applied to address misconceptions about mathematics (see Ref. [46] for a critical review of different intervention approaches for neuromyths). They could also be addressed through structured reflection or by conducting programs designed to change beliefs [43,47,48]. Additionally, it may also be beneficial to implement mindset interventions that could reduce math anxiety [49].

The prevalence of math anxiety among pre-service teachers is a cause for concern. This study found that math anxiety is associated with the belief that mathematical abilities are immutable; furthermore, existing research suggests an association with mathematics teaching anxiety [50,51], as well as with other emotions and attitudes towards the teaching of mathematics [52,53]. Some of these teacher characteristics may negatively impact mathematics teaching and contribute to poorer mathematics learning outcomes in students [54]. Future studies could delve into the origins of these relationships. Additionally, it might be worthwhile to develop educational programs, interventions, or services aimed at reducing math anxiety during teacher training.

In this study, we used a questionnaire created to evaluate myths in mathematics, ensuring continuity in research and facilitating knowledge accumulation. This pioneering questionnaire was developed three decades ago, predating the extensive literature on misconceptions across various domains. Although there is an abundance of literature refuting some myths, updating the research findings that contradict each belief should prove useful.

A potential limitation of this study could lie in the ambiguity of certain items. Depending on the perspective from which an individual approaches a statement, it may be perceived as true or false (see Ref. [55]). For instance, item 4 (math requires a good memory) might be deemed true when considering the numerous studies linking working memory to math performance (for a review, see Ref. [56]). Conversely, if one views mathematics as primarily reliant on memorization, the belief could be considered false, given that mathematics requires other cognitive processes such as reasoning.

## 5. Conclusions

The present study provides some insights into the false math beliefs held by pre-service teachers. First, our results indicate that the prevalence patterns of misconceptions about math are similar to those previously reported in the US across different years. Second, little consistency was found among the math belief items included in the questionnaire, suggesting that diverse origins, rather than a single underlying factor, are likely responsible for the development of different math myths. Finally, some false beliefs about mathematics may predispose individuals to experience negative emotions. Specifically, endorsing certain ideas about who is naturally good at mathematics appears to be linked to math anxiety.

## Figures and Tables

**Table 1:** Summary of the regression analyses of false math beliefs with gender and AMAS and STAI scores as predictors.

	Predictor	β	*SE*	*t*	*P*	95% CI for β
Item 1						
	Gender	0.10	0.14	1.37	0.171	[−0.17, 0.37]
	AMAS	0.17	0.01	2.29	0.023	[0.15, 0.19]
	STAI	0.16	0.01	2.23	0.027	[0.15, 0.17]
Item 7						
	Gender	0.36	0.16	4.98	<0.001	[0.05, 0.66]
	AMAS	0.10	0.01	1.42	0.157	[0.08, 0.12]
	STAI	0.14	0.01	2.01	0.047	[0.13, 0.16]

## Data Availability

Data are available from the corresponding author on reasonable request.

## Appendix A

Items included in the study were taken from Phelps-Gregory et al. [15] and were originally proposed by Kogelman and Warren [21].

1. Some people have a math mind, and some do not. *
2. Math requires logic, not intuition.
3. You must always know how you got the right answer.
4. Math requires a good memory.
5. There is a best way to do a math problem.
6. Math is done by working intensely until the problem is solved.
7. Men are better in math than women. *
8. It is always important to get the answer exactly right.
9. Mathematicians do problems quickly, in their heads.
10. There is a magic key to doing math.
11. Math is not creative.
12. It is bad to count on your fingers.

Note. * indicates specific beliefs related to who can do mathematics.
